# In Vitro Astroglial Dysfunction Induced by Neurotoxins: Mimicking Astrocytic Metabolic Alterations of Alzheimer’s Disease

**DOI:** 10.3390/metabo14030151

**Published:** 2024-03-01

**Authors:** Jéssica Taday, Fernanda Telles Fróes, Marina Seady, Carlos Alberto Gonçalves, Marina Concli Leite

**Affiliations:** Departamento de Bioquímica, Instituto de Ciências Básicas da Saúde, Universidade Federal do Rio Grande do Sul (UFRGS), Porto Alegre 90035-003, Brazil; jessica.taday@ufrgs.br (J.T.); fernanda.froes@ufrgs.br (F.T.F.); pedraseady.marina@mayo.edu (M.S.); marina.leite@ufrgs.br (M.C.L.)

**Keywords:** astrocytes, Alzheimer’s disease, streptozotocin, lipopolysaccharide, fluorocitrate, methylglyoxal

## Abstract

Astrocytes play fundamental roles in the maintenance of brain homeostasis. The dysfunction of these cells is widely associated with brain disorders, which are often characterized by variations in the astrocyte protein markers GFAP and S100B, in addition to alterations in some of its metabolic functions. To understand the role of astrocytes in neurodegeneration mechanisms, we induced some of these metabolic alterations, such as energy metabolism, using methylglyoxal (MG) or fluorocitrate (FC); and neuroinflammation, using lipopolysaccharide (LPS) and streptozotocin (STZ), which is used for inducing Alzheimer’s disease (AD) in animal models. We showed that MG, LPS, STZ and FC similarly caused astrocyte dysfunction by increasing GFAP and reducing S100B secretion. In the context of AD, STZ caused an amyloid metabolism impairment verified by increases in Aβ1-40 peptide content and decreases in the amyloid degradation enzymes, IDE and NEP. Our data contribute to the understanding of the role of astrocytes in brain injury mechanisms and suggest that STZ is suitable for use in vitro models for studying the role of astrocytes in AD.

## 1. Introduction

Astrocytes are implicated in most of the studies of brain disorders accompanied by cognitive impairment, mostly associated with neurodegenerative diseases. Some mechanisms of cognitive impairment are related to astrocytic dysfunction [1,2,3]. Aging, the main risk factor in neurodegenerative diseases [4,5], changes brain metabolism, and studies suggest that cognitive decline can be affected by astroglial metabolism dysfunction [6]. In this sense, considering the accelerated aging of the global population, it is crucial to better understand the role of astrocytes in the neurodegeneration processes.

Astrocytes are glial cells involved in maintaining brain homeostasis and developing synapses and plasticity, contributing to memory consolidation [3,7]. In this sense, it is important to highlight some of their characteristic proteins. S100B is a calcium-binding protein with toxic or trophic functions depending on the context and is considered a marker of brain damage [8]. Glial fibrillary acidic protein (GFAP) is a cytoskeletal protein which is usually associated with the activation of astrocytes [9]. Furthermore, astrocytes play a crucial role in cerebral glutamate metabolism by participating in the glutamate–glutamine cycle, which is related to the cognition process. These cells uptake glutamate from the synaptic cleft and then convert it to glutamine by glutamine synthetase (GS) or use this glutamate to synthesize the endogenous antioxidant glutathione (GSH) [7,10,11]. The disruption of some of these astrocyte functions is associated with the early stages of brain disorders [2,3,12].

Brain degenerative diseases are associated with numerous factors, such as cellular energy metabolism impairment, inflammatory responses and protein aggregation [13]. Many compounds have been used to mimic some of the alterations associated with the initiation or progression of neurodegeneration.

The inhibition of astrocytes by the administration of fluorocitrate (FC), which impairs energy metabolism by the inhibition of aconitase’s impact on the citric acid cycle, specifically in astrocytes [14], has been linked to the inhibition of reactive astrocytes [15,16]. FC administration ameliorated cognitive impairment in models of traumatic brain injury [17] and ischemic stroke [15], highlighting the importance of astrocytes in cognitive damage progression. FC enters the astrocyte via monocarboxylate transporter 1 (MCT1); this lactate transporter is also essential for the brain’s energy metabolism and is crucial for cognition [18]. 

Diabetes mellitus (DM), which leads to cognitive impairment, is a risk factor for neurodegenerative diseases [19]. Methylglyoxal (MG) is a reactive dicarbonyl metabolite of glucose, which is produced during DM and is also used to mimic its features in vitro [20,21,22,23] and in vivo [24,25]. MG leads to an increase in advanced glycation end products (AGEs), which can interact with and alter the levels and activity of their receptor (RAGE) in the brain. [26]. It should be emphasized that astrocytes express RAGE, which, in addition to AGEs [27], binds S100B [28,29] and amyloid beta (Aβ) peptide [30,31]. 

Furthermore, most neurodegenerative diseases and other cognitive impairment conditions are accompanied by neuroinflammation [32]. Lipopolysaccharide (LPS) is a bacterial toxin widely used to induce an inflammatory response by binding to the TLR4 receptor, expressed by glial cells, including astrocytes [33]. In fact, intracerebroventricular injection of LPS causes memory impairment in rats [34]. The TLR4 and RAGE receptors are reportedly altered in neurodegenerative diseases [35,36].

Cognitive impairment and memory loss are the main symptoms of Alzheimer’s disease (AD), the most prevalent neurodegenerative disease, and are frequently related to the presence of characteristic cerebral alterations. Senile plates formed by amyloid beta (Aβ) peptide accumulation and neurofibrillary tangles (NFTs) formed by the hyperphosphorylation of tau protein [37] are considered hallmarks of the disease. However, AD is a multifactorial disease, and other biochemical alterations can start years before the appearance of its symptoms and hallmarks [38]. It is important to note that astrocytic alterations are found in the initial stages of Alzheimer’s disease [12].

Various animal models are available to study AD characteristics, such as intracerebroventricular streptozotocin administration (ICV-STZ). ICV-STZ is a chemical-induced model of sporadic Alzheimer’s disease that has been extensively explored [39,40,41], but the use of STZ in in vitro models has been little explored, especially in astrocytes [42]. The mechanism by which STZ induces AD is not entirely understood, but it is known that the compound can enter CNS cells via glucose transporter 2 (GLUT2) and interfere with the insulin receptor (IR), compromising the glycogen synthase kinase 3 (GSK3) pathway and affecting the metabolism of Aβ peptide and tau phosphorylation. However, the capacity of this compound to interact with astroglial cells needs further investigation [43,44]. Having an appropriate model to study the role of astrocytes in AD is interesting because, even though the production of Aβ by astrocytes is lower than that of neuronal production, considering their high number in the CNS, astrocytic Aβ production is also significant [12]. In addition, astrocytes play an important role in Aβ metabolism by sending it to the peripheral tissues or degrading it by enzymatic mechanisms such as via neprilysin (NEP) or insulin-degrading enzyme (IDE). Therefore, astrocytes can play a dual role in AD; they are protective in the early stages and prevent the accumulation of Aβ peptides; however, upon the progression of the disease, they contribute to the production and accumulation of this peptide [45,46,47].

Astrocytes play a direct role in the protection from and development of neurodegenerative diseases and can be a key point for understanding neurodegenerative disease development, especially AD. However, it is not clear if astrocytic dysfunction induced by different stimuli leads to metabolic alterations observed in AD. Therefore, the objective of the present study was to induce some Alzheimer’s disease-associated dysfunctions in astrocytes by exposing primary astrocyte cultures to MG, FC, LPS or STZ to evaluate the astrocytes’ function and Aβ metabolism. Our results showed that MG, FC, LPS and STZ caused astrocyte dysfunction and suggested that STZ is suitable for use in in vitro models for study of the role of astrocytes in some of the metabolic changes involved with AD.

## 2. Materials and Methods

### 2.1. Materials 

Methylglyoxal (M0252), fluorocitric acid barium salt (F9634), lipopolysaccharides from Escherichia coli 055:B5 (L4005), streptozotocin (S0130), poly-L-lysine (P1274), methyl thiazolyl diphenyl-tetrazolium bromide (MTT) (M2128), neutral red (N4638), propidium iodide (P4170), bisbenzimide H 33258 (14530), amphotericin B solubilized (A9528), HEPES (H7006), sodium bicarbonate (NaHCO3) (S5761), glucose (G5400), albumin from chicken egg white (A5253), phenylmethylsulfonyl fluoride (P7626), ethylene glycol-bis (β-aminoethyl ether)-N,N,N′,N′-tetracetic acid (E4378), dimethyl sulfoxide (D8418), L-glutamic acid γ-monohydroxamate (G2253), anti-S100B antibody (SH-B1) (S2532), S100B protein from bovine brain (S6677), imidazole (I2399), o-phenylenediamine (P9029), reduced glutathione (G4251), phthaldialdehyde (P1378) and L-glutamate (G5889) were purchased from Sigma (St. Louis, MI, USA). Fetal calf serum (FCS) (12657-029), Dulbecco’s modified Eagle’s medium (DMEM) (31600-034), Dulbecco’s phosphate-buffered saline (DPBS) (21600-010) and gentamicin (15710072) were purchased from Gibco (Carlsbad, CA, USA). Cell culture plates were purchased from Falcon (353047 for 24-well, 353043 for 12-well and 353072 for 96-well). Human GFAP (345996) was obtained from Calbiochem. Bovine serum albumin Cohn fraction V (1870) was purchased from Inlab (São Paulo, Brazil). l-[2,3-3 H] Glutamate (ART0103) was purchased from Amersham International (Buckinghamshire, United Kingdom). Anti-neprilysin (AB5458) and anti-IDE (AB9210) antibodies were obtained from Merck Millipore Corporation (São Paulo, Brazil). Anti-Aβ_1-40_ (sc-9129) antibody was obtained from Santa Cruz Biotechnology (Santa Cruz, CA, USA). Polyclonal anti-S100B (Z0311) and anti-GFAP (Z0334) antibodies were purchased from DAKO (São Paulo, Brazil) and anti-rabbit peroxidase (NA934V) and ECL Western blotting detection reagents (RPN3004) were purchased from GE (Little Chalfont, United Kingdom). Rhodamine phalloidin (R415) and acrylamide (15512-023) were purchased from Invitrogen (Waltham, MA, USA). The LDH activity kit (K014-2) was purchased from Bioclin (Belo Horizonte, Brazil). High-binding flat-bottomed plates (655081) used in enzyme-linked immunosorbent assays (ELISAs) were purchased from Greiner Bio-One (Frickenhausen, Germany). All other chemicals were purchased from local commercial suppliers.

### 2.2. Cell Culture

Procedures were carried out in accordance with the National Institutes of Health Guide for the Care and Use of Laboratory Animals and were approved by the Ethics Committee on the Use of Animals of the Federal University of Rio Grande do Sul (number 34855). Primary astrocyte cultures from Wistar rats were prepared as previously described [48]. Briefly, the cerebral cortices of newborn Wistar rats (1−4 days old) were removed and mechanically dissociated in Ca^2+^- and Mg^2+^-free Dulbecco’s phosphate-buffered saline (DPBS), pH 7.4, containing (in mM): 137 NaCl; 2.66 KCl; 8.1 Na_2_HPO_4_; 1.47 KH_2_PO_4_ and 5.55 glucose. After centrifugation at 300× *g* for 5 min, the pellet was suspended in DMEM (pH 7.6) supplemented with 7.68 mM HEPES, 108.79 mM NaHCO_3_, 50 mg/L amphotericin B, 2.5 mg/L gentamicin and 10% fetal calf serum (FCS). Cells were seeded in 12-, 24-, or 96-well plates (800,000, 300,000 and 50,000 cells/well, respectively) pre-coated with poly-L-lysine. Cultures were maintained in DMEM containing 10% FCS in 5% CO_2_/95% air at 37 °C and the medium was changed every 3−4 days. Cells were allowed to grow to confluence and used at 21 days in vitro. We were unable to label neurons or microglia, using anti-NeuN or anti-Iba-1, respectively. 

### 2.3. Experimental Design

**Figure 1:**
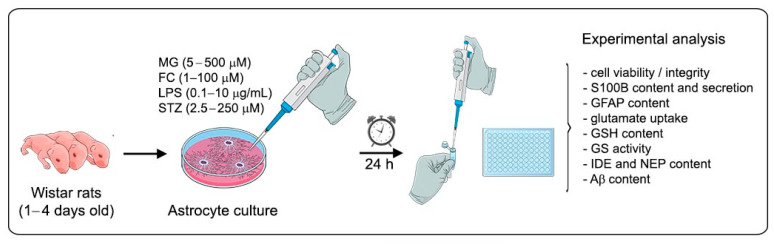
Schematic representation of the experimental plan. Wistar rat astrocytes, cultured after reaching confluence, were exposed to MG (5; 50 or 500 µM), FC (1; 10 or 100 µM), LPS (0.1; 1 or 10 µg/mL), or STZ (2.5; 25 or 250 µM) for 24 h. After incubation, culture medium or intracellular lysate samples were collected for experimental analysis.

The culture medium was replaced by DMEM without FCS (to avoid methodological interference from FCS) in the absence or presence of MG (from 5 to 500 µM), FC (from 1 to 100 µM), LPS (from 0.1 to 10 µg/mL), or STZ (from 2.5 to 250 µM), diluted in DPBS, which were used as the vehicles. The concentration range for each compound was chosen based on previous studies [23,49,50,51]. After 24 h of incubation, the extracellular medium was collected and cell scraping was used to collect the cells, as detailed in each methodological description. A schematic representation of the experimental procedure is shown in Figure 1. 

### 2.4. Methyl Thiazolyl Diphenyl-Tetrazolium Bromide Assay

Cells were incubated with 0.5 mg/mL methyl thiazolyl diphenyl-tetrazolium bromide (MTT) during the last 30 min of the incubation. The medium was removed and the MTT formazan crystals formed by MTT reduction were dissolved in DMSO. Absorbance values were measured at 560 and 650 nm using Spetramax I3 equipment, and MTT reduction was calculated using the following formula: [(abs 560 nm)–(abs 650 nm)]. Results were expressed as a percentage of control.

### 2.5. Neutral Red Incorporation Assay

Cells were treated with 50 μg/mL neutral red (NR) for the last 30 min of the incubation. The medium was removed, and the cells were rinsed twice with phosphate-buffered saline (PBS: 50 mM NaCl, 20 mM NaH_2_PO_4_ and 80 mM Na_2_HPO_4_, pH 7.4) for 5 min each time. NR dye taken up by viable cells was extracted with acetic acid/ethanol/water (1/50/49, *v*/*v*). Absorbance values were measured at 560 nm using the Spetramax I3 equipment, and results were expressed as a percentage of control.

### 2.6. Propidium Iodide Uptake Assay

Cells were treated with 7.5 μM propidium iodide (PI) for the last 15 min of incubation. Fluorescence was measured using 625 nm (excitation) and 713 nm (emission) in Spetramax I3 equipment. Results were expressed as a percentage of control.

### 2.7. Assessment of Lactate Dehydrogenase Activity

The extracellular enzymatic activity of lactate dehydrogenase (LDH) was measured with a commercial assay from Bioclin (Belo Horizonte, Brazil). Briefly, 50 μL of the extracellular medium was transferred to a 96-well plate. The substrate solution containing pyruvate and a solution containing NADH were added to the sample. The catalytic concentration was determined by the speed of decomposition of NADH, measured by the drop in absorbance at 340 nm during 4 min of testing using the Spetramax I3 equipment. Results were expressed as a percentage of control.

### 2.8. Cytochemistry for Actin and Nuclei

The cells were fixed with 4% paraformaldehyde in PBS for 20 min, rinsed with PBS and permeabilized with 0.1% Triton X-100 in PBS (same composition as used in 2.5), for 10 min at room temperature. Afterward, to stain actin, the cells were incubated for 20 min with 2.5 U/mL rhodamine phalloidin, washed with 0.2% Triton X-100 in PBS, and then, nuclei were stained by 100 μM bisbenzimide for 10 min. Cells were visualized using an Olympus CKX41 Inverted Fluorescence Microscope, and representative images were captured.

### 2.9. S100B Measurement 

The medium was collected (for S100B secretion) and the cells were scraped and homogenized in PBS (same composition as used in 2.5), containing 1 mM PMSF (phenylmethylsulfonyl fluoride) and 1 mM EGTA (ethylene glycol-bis (β-aminoethyl ether)-N,N,N′,N′-tetracetic acid) with a syringe and needle (0.3 mm). S100B was measured by ELISA, as previously described [52]. Briefly, 50 μL of the sample or standard (diluted in 0.2% albumin from chicken egg in PBS) plus 50 μL of 50 mM tris buffer were incubated for 2 h at 37 °C on a microtiter plate previously coated overnight with monoclonal anti-S100B (diluted in carbonate buffer) at 4 °C and blocked for 1 h with 2% albumin from chicken egg in PBS at room temperature. Polyclonal anti-S100 (diluted in 0.5% albumin from chicken egg in PBS) was incubated for 30 min at 37 °C, and then, peroxidase-conjugated anti-rabbit antibody (diluted in 0.5% albumin from chicken egg in PBS) was added for 30 min at 37 °C. After incubating o-phenylenediamine for 30 min in the dark at room temperature, the colorimetric reaction was stopped with 3 M HCl and measured at 492 nm, using Spetramax I3 equipment. Data were compared with those of a standard curve (from 0.002 to 1 ng/mL) and intracellular S100B was normalized by the total protein content. Results are expressed as a percentage of control.

### 2.10. Glial Fibrillary Acidic Protein Measurement

The medium was removed, and the cells were scraped and homogenized in PBS containing 1 mM PMSF and 1 mM EGTA with a syringe and needle (0.3 mm). GFAP was measured by ELISA, as previously described [53]. Briefly, 50 μL of standard or cell homogenate sample (diluted in 1 ng/µL bovine serum albumin) was incubated in a microtiter plate overnight at 4 °C and blocked for 2 h with 5% fat-free milk powder tris buffer solution (0.03 M 20 mM tris–HCl, pH 7.5, 0.5 M 137 mM NaCl) (M-TBS). Polyclonal anti-GFAP was incubated for 1 h at room temperature and then peroxidase-conjugated anti-rabbit antibody was added for 1 h at room temperature. After incubating with o-phenylenediamine for 30 min in the dark at room temperature, the colorimetric reaction was stopped with 3 M HCl and measured at 492 nm using the Spetramax I3 equipment. Data were compared with those of a standard curve (from 0.1 to 5 ng/mL) and normalized by the total protein content. Results were expressed as a percentage of control.

### 2.11. Glutamate Uptake Assay

Glutamate uptake was measured, as previously described by [54] with some modifications [55]. Briefly, cell cultures were incubated at 37 °C in Hank’s balanced salt solution (HBSS), containing (in mM) 137 NaCl, 5.36 KCl, 1.26 CaCl_2_, 0.41 MgSO_4_, 0.49 MgCl_2_, 0.63 Na_2_HPO_4_·7 H_2_O, 0.44 KH_2_PO_4_, 4.17 NaHCO_3_ and 5.6 glucose, pH 7.2. The assay was started by adding 0.1 mM L-glutamate and 0.05 μCi/mL L-[2,3-3 H] glutamate. Incubation was stopped after 7 min by removing the medium and rinsing the cells three times with ice-cold HBSS. The cells were then lysed in a 0.5 M NaOH solution. Sodium-independent uptake was determined using N-methyl-D-glucamine instead of NaCl in the HBSS. Sodium-dependent glutamate uptake was obtained by subtracting the non-specific uptake from the total uptake to obtain the specific uptake. Radioactivity was measured in a PerkinElmer Tri-Carb 2300TR scintillation counter. Results were calculated and normalized by the total protein content and are expressed as a percentage of the control.

### 2.12. Reduced Glutathione Content Assay 

The medium was removed, and the cells were scraped and homogenized in KCl phosphate buffer (140 mM, 20 mM, pH 7.4) using a syringe and needle (0.3 mm). Reduced glutathione (GSH) content was measured as previously described by [56]. Briefly, cell homogenates or standard GSH solution (0.977–500 µM) were diluted in sodium phosphate buffer (0.1 M, pH 8.0) containing 5 mM EDTA, and proteins were precipitated with 1.7% meta-phosphoric acid. The supernatant was incubated with o-phthaldialdehyde (1 mg/mL methanol) at room temperature for 15 min in a 96-well black microtiter plate. Fluorescence was measured using excitation and emission wavelengths of 350 and 420 nm, respectively, in the Spetramax I3 equipment. Data were compared with those of the standard curve, normalized by the total protein content and expressed as a percentage of the control.

### 2.13. Glutamine Synthetase Activity

The medium was removed, and the cells were scraped in 50 mM imidazole. The enzymatic activity of glutamine synthetase (GS) was measured as previously described by [57], with modifications. Briefly, cell homogenates were diluted in 50 mM imidazole and incubated in the following manner: with (in mM) 50 imidazole, 50 hydroxylamine, 100 L-glutamine, 25 sodium arsenate dibasic heptahydrate, 0.2 ADP, 2 manganese chloride, pH 6.2 for 15 min at 37 °C. The reaction was stopped by adding 0.37 M FeCl_3_, 0.67 M HCl and 200 mM C_2_HCl_3_O_2_. After centrifugation, the absorbance of the supernatant was measured at 540 nm using the Spetramax I3 equipment and compared to the absorbance generated by standard quantities of L-glutamic acid γ-monohydroxamate diluted in the same solution as the samples. Data were normalized by the total protein content and expressed as a percentage of the control.

### 2.14. Western Blot Analysis 

The medium was removed and the cells were scraped and homogenized in a sample buffer (0.0625 M tris–HCl, pH 6.8, 2% (*w*/*v*) SDS, 5% (*w*/*v*) β-mercaptoethanol, 10% (*v*/*v*) glycerol, 0.002% (*w*/*v*) bromophenol blue), boiled and then centrifuged at 10,000× *g* for 5 min. Equal amounts (15 μg) of total protein were electrophoresed in a 12% (*w*/*v*) SDS–polyacrylamide gel at 180 V for 1 h. The separated proteins were blotted onto a nitrocellulose membrane at an amperage of 1.2 mA/cm^2^ membrane area. Membranes were incubated in 0.05 tween TBS (same composition as used in 2.9) (T-TBS), containing 5% fat-free milk powder diluted in TBS (*w*/*v*) for 1 h at 4 °C. The membranes were incubated overnight at 4 °C with the appropriate primary antibody (diluted 1:5000 in 2.5% bovine serum albumin). Afterward, membranes were incubated overnight at 4 °C with peroxidase-conjugated anti-rabbit antibody (dilution 1:10,000 in 2.5% bovine serum albumin). Equivalent loading of each sample was confirmed with 0.5% India ink dye (in T-TBS containing 1% acetic acid) for 3 h [58]. The chemiluminescence signal was detected using an ECL kit from Amersham and evaluated in the luminescence image analyzer (Image Quant LAS4000 from GE). The luminescence signals were analyzed using ImageJ software, and the optical density was normalized by India ink optical density values. Results were expressed as a percentage of control.

### 2.15. Protein Content

Protein content was determined by Lowry’s method modified by Peterson, using bovine serum albumin as a standard [59]. This method was used to measure total protein content for S100B, GFAP, glutamate uptake, GS and GSH assays.

### 2.16. Statistical Analysis

All analyses were carried out on a PC-compatible computer using SPSS software version 20.0. Parametric or non-parametric tests were used according to the sample number, and the normal distribution and homogeneity of variances were performed by the Shapiro–Wilk and Levene tests, respectively. When normal distribution and homogeneity were assumed, one-way analysis of variance (ANOVA), followed by Tukey post hoc testing, was performed to analyze 3 or more experimental groups. When homogeneity or normal distribution was not assumed, the non-parametric Mann–Whitney test was performed to analyze 2 experimental groups, or the Kruskal–Wallis test followed by pairwise comparison (Dunn–Bonferroni) to analyze 3 or more experimental groups. The statistical analyses are indicated in each of the figure legends. Significance was considered when *p* < 0.05. 

## 3. Results

### 3.1. MG, FC, LPS and STZ Do Not Compromise Cell Viability, Integrity and Morphology 

**Figure 2:**
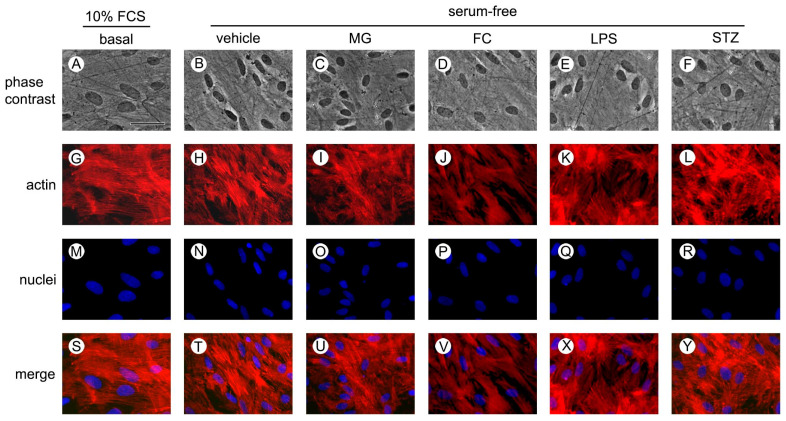
Morphological evaluation of astrocytes. Astrocytes were fixed before (10% FCS DMEM) or after (serum-free DMEM) exposure to 500 µM MG; 100 µM FC; 10 µg/mL LPS or 250 µM STZ for 24 h for visualization. Representative images of phase contrast (**A**–**F**), actin staining with rhodamine phalloidin in red (**G**–**L**), nuclei staining with bisbenzimide in blue (**M**–**R**) and merged images (**S**–**Y**). Scale bar = 50 μm (shown in (**A**)). DMEM, Dulbecco’s modified Eagle’s medium; FCS, fetal calf serum; FC, fluorocitrate; LPS, lipopolysaccharide; MG, methylglyoxal; STZ, streptozotocin.

To evaluate whether MG, FC, LPS or STZ, compounds that mimic some conditions involved in brain injury mechanisms, cause cell damage, cultured astrocytes were exposed to 500 µM MG, 100 µM FC, 10 µg/mL LPS or 250 µM STZ for 24 h before the assays. None of the compounds caused loss of cell viability, as evaluated by the MTT reduction and NR incorporation assays, or integrity, as assessed by measuring PI uptake and LDH extracellular activity. Interestingly, 100 µM (F [4, 40] = 17.903, *p* < 0.001) FC reduced LDH extracellular activity (Table 1). To evaluate if the compounds cause morphological changes, we analyzed the actin filaments assessed by the rhodamine phalloidin assay. After 24 h of exposure to 500 µM MG, 100 µM FC, 10 µg/mL LPS or 250 µM STZ, astrocytes maintained the polygonal shape and organization of actin filaments compared to the vehicle and to the culture’s morphological aspect before the treatment (basal) (Figure 2).

### 3.2. MG, FC, LPS and STZ Affect Astrocyte Protein Markers

**Figure 3:**
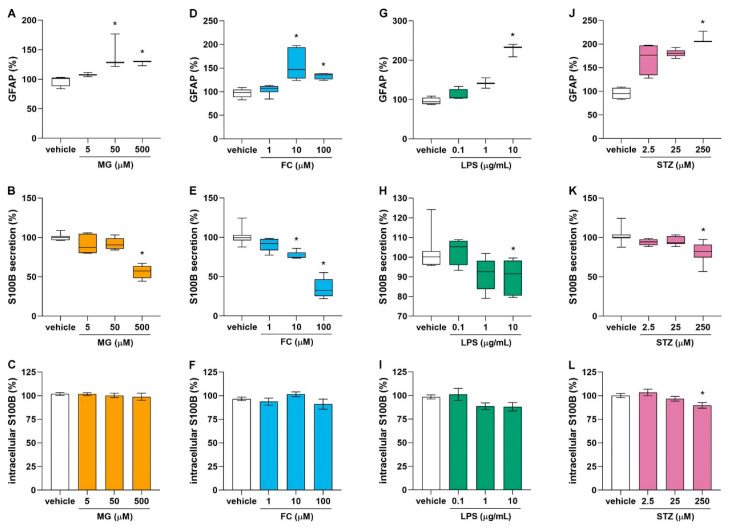
Effects of MG, FC, LPS and STZ on GFAP content, S100B secretion and S100B content. GFAP intracellular content, S100B secretion and S100B content were measured by ELISA in the astrocytes culture after 24 h of exposition to (**A**–**C**) MG (from 5 to 500 µM) (n = 3–7), (**D**–**F**) FC (from 1 to 100 µM) (n = 5–6), (**G**–**I**) LPS (from 0.1 to 10 µg/mL) (n = 3–8) or (**J**–**L**) STZ (from 2.5 to 250 µM) (n = 3–10). Statistical analysis was performed with the Kruskal–Wallis test followed by pairwise comparison (Dunn–Bonferroni) for (**A**,**B**,**D**,**E**,**G**,**H**,**J**,**K**) or one-way ANOVA followed by Tukey post hoc for (**C**,**F**,**I**,**L**). Data are presented as box-and-whiskers plots, where the bottom and the top of the box represent the min to max and the horizontal line is the median (when Kruskal–Wallis was applied). Data are presented as mean ± standard error (when ANOVA was applied). * indicates difference from vehicle. ANOVA, analysis of variance; FC, fluorocitrate; GFAP, glial fibrillary acidic protein; LPS, lipopolysaccharide; MG, methylglyoxal; STZ, streptozotocin.

S100B and GFAP are characteristic astrocyte proteins and can be altered by numerous pathological conditions. We evaluated the intracellular contents of GFAP and S100B, as well as the S100B secretion from cultured astrocytes after 24 h of exposure to different concentrations of MG (from 5 to 500 µM), FC (from 1 to 100 µM), LPS (from 0.1 to 10 µg/mL) or STZ (from 2.5 to 250 µM). These compounds caused a similar response profile of the astrocytes in terms of the S100B secretion and the contents of S100B and GFAP. We observed that 50 µM (H[3] = 12.158, *p* = 0.031) and 500 µM (H[3] = 12.158, *p* = 0.022) MG increased GFAP content (Figure 3A), and 500 µM (H[3] = 13.280, *p* = 0.002) MG caused a reduction in S100B secretion (Figure 3B). For FC, 10 µM (H[3] = 16.856, *p* = 0.002) and 100 µM (H[3] = 12.158, *p* = 0.034) (Figure 3D) increased GFAP content, and also 10 µM (H[3] = 22.916, *p* = 0.040) and 100 µM (H[3] = 22.916, *p* < 0.001) FC caused a reduction in S100B secretion (Figure 3E). For LPS, 10 µg/mL (H[3] = 11.507, *p* = 0.009) (Figure 3G) increased GFAP content, and S100B secretion was found to be reduced by 10 µg/mL (H[3] = 13.789, *p* = 0.032) LPS (Figure 3H). For STZ, the GFAP content was increased by 250 µM (H[3] = 12.525, *p* = 0.003) STZ (Figure 3J), 250 µM (H[3] = 17.489, *p* < 0.001) STZ caused a reduction in S100B secretion (Figure 3K), and only 250 µM (F[3, 22] = 4.470, *p* = 0.040) STZ caused a decrease in the astrocytes’ S100B content (Figure 3L).

### 3.3. MG, FC, LPS and STZ Affect the Glutamate Metabolism of Astrocytes

**Figure 4:**
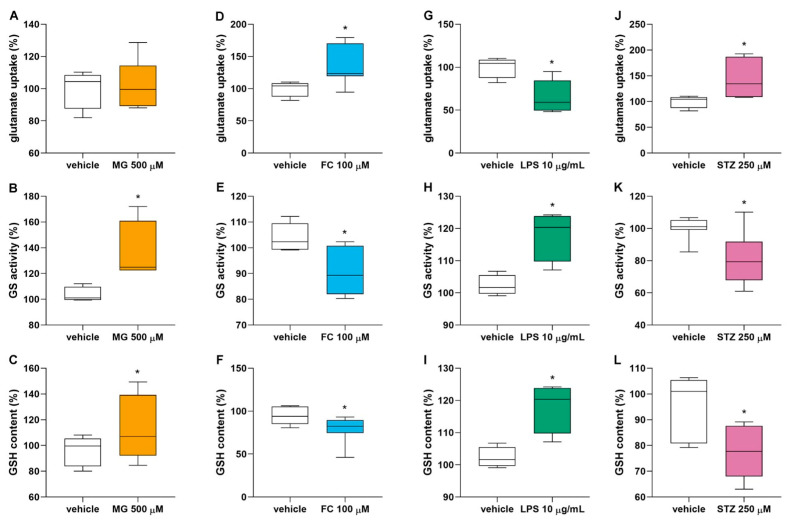
Effects of MG, FC, LPS and STZ on astrocytic glutamate metabolism. Glutamate uptake, GS activity and GSH content were measured in the astrocytes culture after 24 h of exposition to (**A**–**C**) 500 µM MG (n = 3–11), (**D**–**F**) 100 µM FC (n = 5–7), (**G**–**I**) 10 µg/mL LPS (n = 4–5) or (**J**–**L**) 250 µM STZ (n = 5–8). Statistical analysis was performed with the Mann–Whitney test. Data are presented as box-and-whiskers plots, where the bottom and the top of the box represent the min to max, and the horizontal line is the median. * indicates difference from vehicle. FC, fluorocitrate; GS, glutamine synthetase; GSH, reduced glutathione; LPS, lipopolysaccharide; MG, methylglyoxal; STZ, streptozotocin.

Astrocytes have the important function of removing and metabolizing glutamate from the synaptic cleft, in addition to producing GSH. To evaluate whether this important function of astrocytes is affected by different biochemical conditions associated with the brain injury process, we evaluated the glutamate uptake, GSH content and GS activity in astrocytes exposed to 500 µM MG, 100 µM FC, 10 µg/mL LPS, or 250 µM STZ for 24 h (Figure 4). MG (Figure 4A) did not affect astrocytic glutamate uptake, but increased GS activity (U < 0.001, *p* = 0.021) (Figure 4B) and GSH content (U = 30.000, *p* = 0.045) (Figure 4C). FC increased glutamate uptake (U = 5.000, *p* = 0.013) (Figure 4D), whereas it decreased GS activity (U = 3.000, *p* = 0.047) (Figure 4E) and GSH content (U = 17.000, *p* = 0.025) (Figure 4F). LPS decreased glutamate uptake (U = 2.000, *p* = 0.012) (Figure 4G), whereas it increased GS activity (U < 0.001, *p* = 0.021) (Figure 4H) and GSH content (U < 0.001, *p* = 0.021) (Figure 4I). STZ increased glutamate uptake (U = 3.000, *p* = 0.019) (Figure 4J), whereas it decreased GS activity STZ (U = 0.000, *p* = 0.016) (Figure 4K) and (U = 6.000, *p* = 0.018) (Figure 4L).

### 3.4. Only LPS and STZ Affect Proteins Involved in the Amyloid Cascade

**Figure 5:**
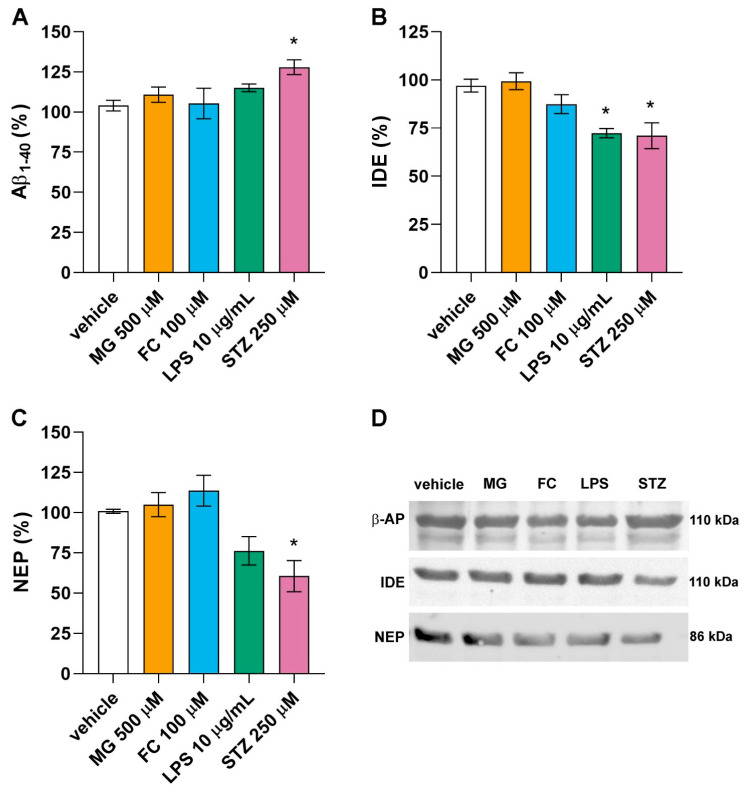
Effects of MG, FC, LPS and STZ on amyloid metabolism. Astrocyte cultures were exposed to 500 µM MG; 100 µM FC; 10 µg/mL LPS or 250 µM STZ for 24 h to evaluate the intracellular content of (**A**) Aβ1-40 (n = 4–6), (**B**) IDE (n = 4) and (**C**) NEP (n = 4–5). In (**D**), representative images of immunoblots. Statistical analysis was performed with one-way ANOVA followed by Tukey post hoc. Data are presented as mean ± standard error. * indicates difference from vehicle. Aβ1-40, amyloid beta; ANOVA, analysis of variance; FC, fluorocitrate; IDE, insulin-degrading enzyme; LPS, lipopolysaccharide; MG, methylglyoxal; NEP, neprilysin; STZ, streptozotocin.

Astrocytes are involved in early alterations observed in neurodegenerative diseases, such as AD. To evaluate the role of astrocytes in the amyloid cascade, under our experimental conditions, we evaluated some of the proteins involved in the Aβ accumulation process (Aβ1-40, IDE and NEP) in astrocytes exposed to 500 µM MG, 100 µM FC, 10 µg/mL LPS, or 250 µM STZ for 24 h. Only LPS and STZ altered the content of these proteins. LPS decreased the astrocytes’ content of IDE (F[4, 15] = 8.420, *p* = 0.013) (Figure 5B). STZ increased the Aβ1-40 peptide content (F[4, 21] = 3.039, *p* = 0.036) (Figure 5A), accompanied by a decrease in the content of the amyloid degradation enzymes, IDE (F[4, 15] = 8.420, *p* = 0.009) (Figure 5B) and NEP (F[4, 16] = 7.177, *p* = 0.026) (Figure 5C).

## 4. Discussion

Astrocytes play a crucial role in maintaining brain homeostasis in the CNS, and dysfunction of astrocytes has been reported during the early phases of some brain disorders [3,46], emphasizing the importance of understanding the roles of astrocytic alterations in this context. Biochemical and metabolic alterations that cause or contribute to the development of cognitive impairment include glucose homeostasis disruption, neuroinflammation and the formation of protein aggregates [13]. As such, we selected four different compounds to mimic some important characteristics associated with the initiation or progression of cognitive disorders, aiming to study some astrocytic metabolic features within this context: MG (used to mimic high glucose and DM in vivo), FC (an inhibitor of energy metabolism), LPS (used to induce neuroinflammation), and STZ (used to induce AD in animal models).

To choose the concentration of use of the compounds, we first exposed the astrocyte cultures to different concentrations of MG, FC, LPS or STZ and evaluated the level of GFAP and S100B, which are frequently associated with brain disorders [9,60]. In fact, they seem to be an essential astrocytic response mechanism, as all of the stimuli tested on the astrocytes induced the same response profile: an increase in astrocytic GFAP levels and a decrease in S100B secretion without evidence of cell damage. A previous study from our group showed that hippocampal slices exposed to MG presented diminished S100B secretion; furthermore, in vivo, MG ICV administration modulated S100B in the short term (72 h) [25] but not over a more extended period (6 weeks) [24]. With regard to FC, reductions in S100B secretion have previously been observed in hippocampal slices and astrocyte cultures after 1 h of exposure [50]. We found that this effect persists until 24 h and that astrocytes also exhibited increased GFAP. Interestingly, in animal models, FC reduced the elevation in GFAP caused by LPS in a depression-like model [61]. Corroborating our results, several studies have described the effects of LPS on S100B and GFAP in isolated astrocytes [49,62,63,64,65]. In STZ-induced AD, the increase in GFAP brain levels precedes Aβ accumulation and NFT formation [39,41,66]. Similarly, S100B, considered a cerebral damage marker, is increased in AD patients’ serum, whereas its levels in the CSF demonstrate discrepant results (see Gonçalves et al., 2008 for a review). On the other hand, in STZ-induced AD, S100B is decreased in the CSF and elevated in the hippocampus within a short time after injection (one week) and increased in the CSF and decreased in the hippocampus after 4 weeks [67]. Similarly, in acute hippocampus slices, S100B was reduced by STZ after 1 h [41]. Our data corroborated these findings in cultured astrocytes. The significance of cerebral GFAP and S100B levels should be discussed according to the context [68]; here, considering situations involved with brain damage, all of the compounds tested showed a similar profile, suggesting astrocytic dysfunction, as found in neurodegenerative diseases.

Based on the effects observed on S100B and GFAP, we investigated astrocytic function, assessing some features related to glutamate and Aβ metabolism by the highest concentration of MG, FC, LPS or STZ. Astrocytes play an important role in glutamate detoxification via the glutamate–glutamine cycle, and dysfunctions in this system have been observed in cognitive disorders [10,69]. Mimicking a DM2 condition in astrocytes, MG increased GSH content and GS activity without affecting the glutamate uptake. This could represent a protective mechanism in astrocytes from MG, as previously observed in an astrocyte culture incubated with 1 mM MG, which was found to cause increases in GSH and glyoxalase 1 enzyme in association with a decrease in glutamate uptake [23]. The most prevalent type of dementia is AD, characterized, among other alterations, by Aβ accumulation due to an imbalance in its production and clearance. Aβ clearance by astrocytes plays a critical role in AD development, and a decreased rate of Aβ clearance has been reported in AD patients [70,71]. As such, astrocytes express enzymes that degrade Aβ, such as NEP and IDE [47,72]. Alterations in these enzymes during the early stages of AD have been reported as crucial to disease progression [45,47]. Glycation reactions are suggested to enhance Aβ formation pathways in amyloid precursor protein (APP) processing mechanisms [73], and the clearance of Aβ by astrocytes was impaired after inhibiting insulin signaling pathways, in turn reducing NEP and IDE levels [74]. Despite that, the alterations in glycation induced by MG in our conditions did not impact these targets involved in the Aβ metabolism.

The inhibition of energy metabolism by FC had a different profile regarding glutamate metabolism. In this scenario, we observed an increase in glutamate uptake, a decrease in GSH content and no change in GS enzyme activity. Previous studies showed an impairment of glutamate metabolism, leading to reductions in glutamate uptake and glutamine synthesis [75,76]. Paulsen and co-workers observed a similar result after 4 h of an intrastriatal administration of FC, which was reversed in 24 h [77]. Energy hypometabolism is found in AD patients, and evidence suggests this is important at the beginning of AD [78]. Although the effects of FC on AD are not entirely understood, in an animal model of AD induced by Aβ1-42 injection in the hippocampus, FC was found to reduce the acquisition of spatial memory [79] and also increased tau phosphorylation in the hippocampus [80]. Regarding Aβ-metabolism and its accumulation, similarly to MG, FC did not cause any alteration in our conditions.

Inflammation is a crucial mechanism shared by many neurodegenerative diseases. The excitotoxicity and the increase in Ca^+2^ influx in the neural cells may be caused by an impaired glutamate uptake by astrocytes. Accordingly, we observe that LPS-induced inflammation decreased glutamate uptake, suggesting an accumulation of glutamate in the synaptic cleft possibly leading to excitotoxicity, as shown in neuronal cell cultures, hippocampus slices and cerebral cortex tissue [81,82,83]. Further, we found an increase in GS enzyme activity and no alterations in GSH content in astrocytes. A decrease in GSH content was observed in cultured astrocytes exposed to LPS (from 0.01 to 30 µg/mL, except at 1 µg/mL) for 24 h [49]. Similarly, hippocampus slices exposed to 10 µM/mL LPS for 6 h showed a reduction in glutamate uptake and GSH content [83]. Different from what we observed for MG and FC, LPS induced a decrease in IDE protein expression in astrocytes without changes in Aβ intracellular content. Indeed, neuroinflammation is reported to represent a critical alteration in AD development [84,85]. The role of inflammation in the amyloidogenic pathway was indicated in astrocytes by the increased expressions of proteins such as APP and BACE [86]. We demonstrated that LPS is also involved in AD development by impairing the Aβ clearance mechanisms of astrocytes before the accumulation of Aβ.

ICV injection of STZ is widely used as a model of AD in animals, although most in vitro models have explored its effect on neurons [51,87,88]. The mechanisms by which STZ induces AD development are not fully understood, but it has been proposed that this compound can enter neurons through GLUT2 and affect the insulin receptor, thereby improving the GSK3 pathway [89]. After exposing the astrocytes to STZ, we found an increase in glutamate uptake, but decreased GSH levels and GS activity. Indeed, the effects of STZ on GSH and GS have been extensively shown in animal models from 1 to 8 weeks after STZ administration [43,68,90]. In this work, we showed, for the first time, that these effects are directly related to astrocyte function. An in vivo study measured glutamate uptake in hippocampal slices 4 weeks after STZ administration, and it was not altered. Notably, at this stage, the animal already shows some late alterations consistent with AD, such as cognitive damage [90]. In isolated astrocytes, an increase in glutamate uptake was observed at 24 h, probably indicating a compensatory mechanism during the early stages. Regarding Aβ metabolism, STZ was the only compound tested in this work that caused a reduction in IDE and NEP protein contents associated with an increase in Aβ1-40 content. In previous work using neuronal primary culture, 8 mM STZ increased the mRNA expressions of APP, MAPT, GSK3α and GSK3β at 24 h; all of these pathways are involved in Aβ production and tau phosphorylation [87]. Herein, we showed that STZ could directly affect astrocytic mechanisms involved in AD, such as clearance by NEP and IDE, and therefore contribute to Aβ accumulation.

It is worth highlighting that the profile of alterations caused by the model using STZ ICV injection to induce AD in animals was reproduced in isolated astrocytes in this study regarding GFAP, S100B, GS and GSH measurements. In addition, our data related to amyloid metabolism showed a direct response of astrocytes to STZ in the AD context. Together, these findings suggest that the use of STZ is appropriate for the study of the role of astrocytes in AD in an in vitro model.

## 5. Conclusions

We showed that high glucose concentrations, astrocyte inhibition, neuroinflammation and AD development characteristics mimicked by MG, FC, LPS and STZ caused astrocyte activation and decreased S100B secretion. However, the insults used affected the glutamate metabolism of astrocytes differently, and only STZ reduced both the Aβ degrading enzymes, NEP and IDE, and caused elevations in Aβ levels. Thus, all of the compounds affect astrocytes, altering cellular functions and contributing to the progression of brain disorders such as AD, but STZ seems to provide a better model to study the role of astrocytes in some features of AD in vitro.

## Figures and Tables

**Table 1:** Effects of MG, FC, LPS and STZ on cell viability and integrity.

	MG 500 μM	FC 100 μM	LPS 10 μg/mL	STZ 250 μM
MTT	102.5 ± 3.3 (*p* = 1)	102.9 ± 3.3 (*p* = 0.645)	99.4 ± 2.1 (*p* = 0.706)	102.8 ± 2.0 (*p* = 1)
NR	101.1 ± 2.7 (*p* = 1)	107.1 ± 1.1 (*p* = 0.999)	101.8 ± 3.6 (*p* = 1)	100.9 ± 4.0 (*p* = 0.844)
PI	103.5 ± 2.4 (*p* = 1)	89.1 ± 3.7 (*p* = 0.074)	97.5 ± 3.4 (*p* = 0.768)	98.7 ± 2.2 (*p* = 0.875)
LDH	101.0 ± 5.3 (*p* = 1)	59.4 ± 5.6 (*p* < 0.001) *	96.2 ± 4.5 (*p* = 0.819)	97.1 ± 4.7 (*p* = 0.934)

Statistical analyses were performed with one-way ANOVA followed by Tukey post hoc. Data are presented as mean ± standard error. * indicates difference from vehicle (n = 4−8). ANOVA, analysis of variance; FC, fluorocitrate; LDH, lactate dehydrogenase extracellular activity; LPS, lipopolysaccharide; MG, methylglyoxal; MTT, methyl thiazolyl diphenyl-tetrazolium bromide reduction; NR, neutral red incorporation; PI, propidium iodide uptake; STZ, streptozotocin.

## Data Availability

The raw data supporting the results of this study are, by collective decision of the authors, available from the corresponding author [MC Leite] upon request.
